# Integrative oncology in colorectal cancer: evidence-based strategies from prevention through survivorship

**DOI:** 10.3389/fonc.2026.1860619

**Published:** 2026-06-26

**Authors:** Cailu Shen, Rui Lou, Fan Bai, Ying Zhang, Tong Xu, Zebo Huang

**Affiliations:** 1 Department of Medical Oncology, Affiliated Hospital of Jiangnan University, Wuxi, China; 2 Department of Medical Oncology, The Sixth Affiliated Hospital, Sun Yat-sen University, Guangzhou, China

**Keywords:** chemoprevention, colorectal cancer, dietary interventions, *Fusobacterium nucleatum*, gut microbiome, integrative oncology, survivorship

## Abstract

Colorectal cancer (CRC) is a leading global malignancy, with approximately 1.9 million new cases and over 900,000 deaths recorded in 2022, yet evidence-based integrative oncology strategies remain inconsistently incorporated into routine care. This narrative review synthesizes current evidence across the full CRC care continuum, from primary prevention through long-term survivorship. High-fiber diets, Mediterranean dietary patterns, calcium supplementation, regular physical activity, healthy weight maintenance, and berberine each demonstrate reproducible CRC risk reduction in large prospective cohorts and multicenter randomized controlled trials. The CHALLENGE trial (NEJM 2025) provides the first randomized phase 3 evidence that structured exercise after adjuvant chemotherapy reduces disease recurrence (HR 0.72) and death (HR 0.63) in colon cancer. Aspirin chemoprevention requires individualized risk–benefit assessment per 2022 US Preventive Services Task Force guidelines; preliminary CaPP3 trial data (conference presentation, June 2025; not yet peer-reviewed) suggest non-inferiority of low-dose aspirin (75–100 mg/day) to 600 mg/day in Lynch syndrome, pending formal publication. Fusobacterium nucleatum promotes colorectal carcinogenesis through five mechanistically distinct pathways: FadA-mediated Wnt/β-catenin activation, Fap2- and CbpF-mediated immune evasion via TIGIT and CEACAM1, succinate–HIF-1α–EZH2-mediated immune suppression, Hippo pathway-mediated suppression of pyroptosis, and autophagy-induced chemoresistance. Perioperative multi-strain probiotics significantly reduce postoperative infectious complications, and fecal microbiota transplantation shows preliminary promise for sensitizing microsatellite-stable CRC to immunotherapy. The 2022–2024 SIO–ASCO and ASCO–SIO clinical practice guidelines endorse mindfulness-based interventions, yoga, and acupuncture for anxiety, depression, fatigue, and cancer-related pain. Systematic integration of these interventions into multidisciplinary CRC care requires standardized implementation frameworks, CRC-specific clinical trials for mind-body modalities, and bioavailability-optimized phytochemical formulations.

## 1 Introduction

Colorectal cancer (CRC) ranked third in global cancer incidence and second in cancer-related mortality in 2022, with approximately 1.9 million new cases and more than 900,000 deaths across 185 countries (1). Epidemiological trends are diverging. Incidence has declined in high-income countries with established screening programs, yet it is rising sharply among adults under 50—a shift attributed to Westernized diet and lifestyle—and in low- and middle-income countries undergoing rapid epidemiological transition (2). Global CRC burden is projected to exceed 3.2 million incident cases annually by 2040 (3). These trends create an urgent need for innovation in both preventive and supportive care.

Surgery, systemic chemotherapy, targeted therapy, and immunotherapy have each advanced over the past decade, yet the five-year survival rate for metastatic CRC remains below 15%. Treatment-related toxicities also persist. Oxaliplatin-induced peripheral neuropathy, cancer-related fatigue, and psychological distress impair quality of life at every disease stage (4). These unmet needs have spurred clinical and research interest in integrative oncology, defined as the evidence-informed application of dietary, botanical, microbiome-targeted, and mind-body interventions alongside conventional cancer treatment (5).

The evidence base for integrative CRC care has expanded considerably over the past decade, driven by multicenter randomized controlled trials (RCTs), umbrella reviews, and successive professional society guidelines. An umbrella review of prospective observational meta-analyses confirmed reproducible CRC risk reduction with dietary fiber, Mediterranean dietary patterns, and calcium supplementation (6). The multicenter Chemoprevention of Berberine in Adenoma Recurrence (CBAR) RCT reported significant adenoma recurrence reduction (7). Fusobacterium nucleatum (Fn) has been characterized as a key oncogenic driver through multiple well-defined molecular mechanisms (8). Three successive SIO–ASCO and ASCO–SIO clinical practice guidelines issued between 2022 and 2024 now provide evidence-graded recommendations for integrative interventions across pain, anxiety, depression, and fatigue (9–11). Nevertheless, integrative approaches remain inconsistently applied in routine oncology care (12).

This review examines four integrative domains across the full prevention-to-survivorship continuum: dietary and lifestyle interventions, phytochemical and chemopreventive strategies, microbiome-targeted therapies, and mind-body practices. Particular emphasis is placed on the molecular biology of Fn, perioperative microbiome management, and evidence-based survivorship care. To our knowledge, this is the first narrative review to integrate the five mechanistically distinct Fn carcinogenic pathways with the full spectrum of evidence-based integrative CRC interventions in a single clinical framework. The synthesis is contextualized by the recently discovered Fna C2 clade specificity and incorporates the most recent ASCO–SIO guidelines (2022–2024) and emerging CaPP3 trial data.

Literature was identified by searching PubMed, Web of Science, and the Cochrane Library for publications through 2025. Search terms included “colorectal cancer”, “colon cancer”, and “rectal cancer”, each combined with “diet”, “dietary fiber”, “Mediterranean diet”, “physical activity”, “obesity”, “aspirin”, “berberine”, “curcumin”, “gut microbiome”, “Fusobacterium nucleatum”, “probiotics”, “fecal microbiota transplantation”, “mindfulness”, “yoga”, “acupuncture”, and “integrative oncology”. Priority was given to RCTs, prospective cohort studies, systematic reviews, meta-analyses, and umbrella reviews; mechanistic studies were included where essential for interpreting clinical findings. When multiple systematic reviews or meta-analyses addressed the same clinical question, we prioritized the most recent publication with the largest pooled sample and lowest heterogeneity. Conflicting findings were presented alongside explicit notation of methodological differences that may account for inconsistencies. Studies reporting only preclinical data were excluded except in the Fusobacterium nucleatum mechanistic sections, where preclinical evidence was necessary to construct the pathogenic framework. No formal PRISMA process was applied, consistent with the narrative review format.

## 2 Dietary and lifestyle interventions in CRC prevention

### 2.1 Dietary fiber, calcium, and vitamin D

Diet, physical activity, and body weight are among the most modifiable determinants of CRC risk, exerting their effects through gut microbial metabolite production, systemic inflammation, metabolic hormone signaling, and epigenetic regulation of colonocyte biology. An umbrella review of prospective observational meta-analyses provides the most comprehensive synthesis to date of dietary and lifestyle risk and protective factors for CRC, supporting targeted counseling as a cornerstone of primary and secondary prevention (6).

Dietary fiber lowers CRC risk chiefly through colonic fermentation, which generates short-chain fatty acids (SCFAs). Butyrate, the principal energy substrate for colonocytes, is the most important of these. It inhibits histone deacetylase (HDAC), suppresses NF-κB-driven inflammation, upregulates tight-junction proteins to reinforce mucosal barrier integrity, and restrains neoplastic transformation (13). Pooling 25 prospective cohort studies (n > 2 million participants), Aune et al. estimated approximately 10% lower CRC risk per 10 g/day increment in total dietary fiber intake, with the strongest associations for cereal fiber and whole grains (14). Current evidence supports a target of ≥25–30 g/day. Mean Western intake remains at roughly 15–18 g/day, well below this threshold (15).

Calcium supplementation at 1,000–1,200 mg/day significantly reduces colorectal adenoma recurrence (RR 0.80, 95% CI 0.68–0.93), as documented across one umbrella review and multiple prospective meta-analyses (6, 16). Two complementary mechanisms underlie this protective effect: calcium binds secondary bile acids and ionized fatty acids in the colonic lumen, reducing their cytotoxicity; and calcium-sensing receptor (CaSR) activation on colonocytes promotes differentiation and suppresses aberrant proliferation. Evidence is strongest for adenoma recurrence prevention after polypectomy; primary prevention data remain less conclusive.

Vitamin D modulates the cell cycle, apoptosis, and immune surveillance through vitamin D receptor (VDR)-mediated transcriptional regulation. Mendelian randomization analyses support a causal inverse association between circulating 25-hydroxyvitamin D and CRC risk (OR 0.88 per 25 nmol/L increment, 95% CI 0.81–0.96) (17). The VITAL trial found no statistically significant reduction in CRC incidence with vitamin D3 supplementation (18), a null result likely attributable to heterogeneity in baseline vitamin D status among enrolled participants. Correction of documented deficiency is clinically prudent, while routine high-dose supplementation for CRC prevention is not currently supported by RCT evidence.

### 2.2 Mediterranean diet

The Mediterranean dietary pattern combines multiple chemopreventive components: abundant vegetables, fruits, legumes, whole grains, olive oil, and fish, alongside restricted red meat. Compared with the lowest adherence category, the highest adherence was associated with a 14% lower CRC risk (RR 0.86, 95% CI 0.80–0.94) in a cancer site-specific meta-analysis (19), a finding independently corroborated in a broader synthesis of 117 studies encompassing 3.2 million participants (20). Olive oil polyphenols suppress NF-κB activation; omega-3 fatty acids attenuate prostaglandin E2 (PGE2) synthesis via cyclooxygenase-2 (COX-2); and prebiotic fibers enrich SCFA-producing microbiota. High Mediterranean diet adherence also beneficially reshapes the gut microbial metabolome by increasing SCFAs, reducing secondary bile acids, and lowering trimethylamine N-oxide (TMAO), a pro-inflammatory metabolite independently linked to CRC risk (21).

### 2.3 Red and processed meat and alcohol

Red meat and processed meat are classified by the International Agency for Research on Cancer as Group 2A (probable) and Group 1 (definite) human carcinogens, respectively. Dose-dependent CRC risk elevation has been confirmed across prospective studies: each 100 g/day increment in red meat intake was associated with a 17% higher CRC risk (RR 1.17, 95% CI 1.05–1.31), and each 50 g/day increment in processed meat intake with an 18% higher risk (RR 1.18, 95% CI 1.10–1.28) (22). Underlying mechanisms include N-nitroso compound formation, heme iron-catalyzed lipid peroxidation yielding cytotoxic aldehydes, and heterocyclic amine production during high-temperature cooking.

Heavy alcohol consumption (≥50 g ethanol/day) was associated with a 44% higher CRC risk in heavy drinkers compared with nondrinkers and occasional drinkers (RR 1.44) in a comprehensive dose–response meta-analysis of 572 studies, with a continuous dose–risk relationship across the full range of intake (23). Acetaldehyde, the principal ethanol metabolite, induces DNA double-strand breaks and impairs mismatch repair; alcohol additionally depletes folate, disrupting one-carbon metabolism essential for DNA methylation fidelity.

### 2.4 Physical activity and sedentary behavior

Regular physical activity is one of the best-established modifiable determinants of CRC risk. Wolin et al. documented 24% lower colon cancer risk among the most versus least physically active individuals (summary RR 0.76, 95% CI 0.72–0.81) in a meta-analysis of prospective cohort studies (24). The protective effect is anatomically specific: colon cancer benefits considerably more than rectal cancer. Physical activity lowers CRC risk through reduced circulating insulin and insulin-like growth factor 1 (IGF-1), attenuation of adipokine-driven inflammation, COX-2 downregulation, and acceleration of colonic transit limiting mucosal exposure to luminal carcinogens (24).

The survival benefit of physical activity extends beyond prevention into post-diagnosis care. In a meta-analysis of 21 prospective studies, Schmid and Leitzmann found that physically active CRC survivors had significantly lower CRC-specific mortality (HR 0.61, 95% CI 0.40–0.92) and all-cause mortality (HR 0.68, 95% CI 0.56–0.82) compared with their inactive counterparts (25). Combined aerobic and resistance training produces greater improvements in cardiorespiratory fitness than either modality alone (26). The American College of Sports Medicine recommends ≥150 min/week of moderate-intensity aerobic activity plus two resistance-training sessions per week for cancer survivors, with individualized adaptation for treatment-related functional limitations including stoma management, neuropathy, and pelvic-floor dysfunction (27).

Sedentary behavior is an independent CRC risk factor, distinct from and additive to physical inactivity. A dose–response meta-analysis of 28 prospective cohort studies found that each 2-hour increment in daily sitting time was associated with a 2–3% higher CRC risk, independent of leisure-time physical activity (28). Patients who meet exercise guidelines but remain sedentary for extended periods should still be counseled to interrupt sitting time with regular movement breaks.

The CHALLENGE trial (Canadian Cancer Trials Group CO.21) provides the first randomized phase 3 evidence that structured exercise improves cancer outcomes in colon cancer. This international trial, conducted at 55 centers, enrolled 889 patients with resected stage III or high-risk stage II colon cancer who had completed adjuvant chemotherapy and were exercising less than the equivalent of 150 min/week of moderate-to-vigorous intensity. Patients were randomly assigned to a 3-year structured exercise program (with a goal of increasing recreational aerobic activity by ≥10 MET-hours/week) or to health-education materials alone. After a median follow-up of 7.9 years, disease-free survival was significantly longer in the exercise group (HR 0.72, 95% CI 0.55–0.94; P = 0.02; 5-year DFS 80.3% vs. 73.9%). Results also supported longer overall survival (HR 0.63, 95% CI 0.43–0.94; 8-year OS 90.3% vs. 83.2%), although overall survival was a secondary endpoint and the trial was not formally powered for this comparison (29). The CHALLENGE results provide level 1 evidence that structured exercise after adjuvant chemotherapy reduces recurrence and support its integration into post-adjuvant survivorship care.

### 2.5 Obesity and metabolic syndrome

Excess body adiposity is a causally supported CRC risk factor, with Mendelian randomization studies reinforcing the observational evidence. Drawing on 141 prospective cohort studies, Renehan et al. found that each 5-unit BMI increment conferred a 24% higher colon cancer risk in men (RR 1.24, 95% CI 1.20–1.28) and a 9% higher risk in women (RR 1.09, 95% CI 1.04–1.14), with stronger associations for proximal than for distal colon cancer (30). Waist circumference ≥102 cm in men or ≥88 cm in women independently predicts CRC risk beyond BMI alone, reflecting the disproportionate carcinogenic potential of visceral adiposity (31).

Visceral fat expansion drives chronic low-grade inflammation through elevated TNF-α, IL-6, and leptin. Simultaneously, hyperinsulinemia and elevated IGF-1 activate the PI3K/AKT/mTOR pathway in colonocytes, fostering proliferation while suppressing apoptosis. Reduced adiponectin further removes an important anti-inflammatory and anti-proliferative signal at the colonic epithelium. A modest 5–10% reduction in body weight through dietary modification and physical activity measurably lowers circulating insulin, IGF-1, and pro-inflammatory cytokines (31).

### 2.6 Aspirin chemoprevention

Aspirin exerts chemopreventive activity through irreversible COX-2 inhibition, suppressing tumor-promoting PGE2 synthesis and attenuating downstream pro-angiogenic and pro-proliferative signaling. Long-term follow-up of randomized prevention trials and observational cohorts demonstrated 20–30% lower CRC incidence with regular aspirin use (75–300 mg/day) over ≥10 years (32). The CAPP2 trial provides the strongest evidence in Lynch syndrome. This double-blind, placebo-controlled RCT enrolled 861 Lynch syndrome carriers and confirmed significant CRC risk reduction with 600 mg aspirin daily. The intention-to-treat HR was 0.65 (95% CI 0.43–0.97; p = 0.035), with a per-protocol HR of 0.56 (95% CI 0.34–0.91) among the 509 participants completing ≥2 years of treatment over 10-year follow-up (33).

The subsequent CaPP3 dose non-inferiority trial enrolled 1,879 Lynch syndrome carriers across five countries, randomizing them to 75–100 mg, 300 mg, or 600 mg aspirin daily for five years. Preliminary data presented at the Cancer Research UK International Cancer Prevention Conference (London, 25–27 June 2025)—not yet published in a peer-reviewed journal at the time of this submission—indicated that low-dose aspirin (75–100 mg/day) was non-inferior to 600 mg/day for CRC prevention while carrying a more favorable gastrointestinal safety profile (34). These findings, if confirmed on formal peer-reviewed publication, would provide additional support for routine low-dose aspirin prescribing in Lynch syndrome.

The 2022 USPSTF guideline update revised recommendations markedly for average-risk adults. Initiating aspirin for primary prevention in adults ≥60 years is now advised against (Grade D) owing to an unfavorable hemorrhage risk–benefit balance; individualized assessment is recommended for adults aged 40–59 years with ≥10% ten-year cardiovascular disease risk (Grade C) (35). The most favorable risk–benefit profile for aspirin chemoprevention remains in Lynch syndrome carriers and patients with prior advanced adenoma.

## 3 Phytochemicals in CRC prevention

### 3.1 Berberine

Plant-derived bioactive compounds modulate multiple oncogenic signaling pathways, making them candidates for CRC chemoprevention with generally acceptable safety profiles. Clinical translation has been uneven: preclinical efficacy does not reliably predict human trial outcomes, and limited oral bioavailability has impeded development of several promising candidates. Berberine is uniquely supported by multicenter randomized trial evidence; curcumin, despite extensive mechanistic characterization, has not translated laboratory promise into clinical benefit.

Berberine, an isoquinoline alkaloid derived from Berberis species, is the only phytochemical with demonstrated CRC chemoprevention efficacy in a multicenter RCT. The CBAR trial was a double-blind, placebo-controlled study conducted at seven hospital centers across six provinces in China. It showed that berberine 0.3 g twice daily significantly reduced colorectal adenoma recurrence over two years in patients with prior adenoma resection (36% vs 47%; RR 0.77, 95% CI 0.66–0.91; p = 0.001; number needed to treat ≈ 9) (7). A subsequent 6-year follow-up of the trial cohort reported continued reductions in adenoma recurrence and neoplasm occurrence; this extended follow-up was, however, a retrospective observational analysis of the original participants rather than a continuation of randomized, double-blind, placebo-controlled treatment, and therefore suggests—rather than confirms—a sustained chemopreventive effect (36). Mild gastrointestinal symptoms were the predominant adverse effect.

Beyond adenoma recurrence prevention, berberine exerts complementary antiproliferative and microbial effects that together underpin its chemopreventive activity. AMPK activation suppresses mTOR-driven colonocyte proliferation and improves insulin sensitivity; concurrent COX-2 inhibition attenuates PGE2-mediated tumor-promoting inflammation; and preclinical data show selective reduction of oncogenic gut taxa alongside enrichment of SCFA-producing commensals (37). This convergence of direct antiproliferative signaling with microbiome remodeling sets berberine apart from agents targeting a single molecular node. International confirmatory trials in non-Chinese populations with extended follow-up remain a priority before broad guideline endorsement. Until such data are available, berberine may be considered for secondary prevention after adenoma resection, with appropriate counseling regarding the current geographic limitation of the evidence base.

### 3.2 Curcumin

Curcumin inhibits NF-κB, Wnt/β-catenin, STAT3, and EGFR signaling pathways implicated in CRC carcinogenesis (38). An early proof-of-concept signal emerged from a small, uncontrolled pilot study (n = 5) in familial adenomatous polyposis (FAP), which reported 60.4% polyp reduction following combined treatment with curcumin and quercetin (39). This finding carries no inferential validity for curcumin as a single agent: the study had no control arm and used two distinct compounds concurrently. The principal obstacle to clinical translation is pharmacokinetic: oral bioavailability remains below 1% owing to rapid phase II metabolism (40). A randomized phase IIa safety trial of curcumin 2 g/day combined with FOLFOX chemotherapy in metastatic CRC observed no meaningful progression-free survival signal (41). Nanoparticle, liposomal, and phospholipid-complex formulations improve curcumin pharmacokinetics in early-phase studies but await CRC-specific clinical validation (42). Curcumin therefore remains investigational, and its clinical adoption will depend on bioavailability-optimized formulation development and adequately powered phase II/III CRC-specific RCTs.

## 4 The gut microbiome in CRC pathogenesis and treatment

### 4.1 Microbiome dysbiosis and CRC risk

The gut microbiome shapes CRC risk, prognosis, and treatment response through immune modulation, microbial metabolite production, and direct pharmacological interactions with anticancer agents.

The human gut harbors approximately 3.8×1013 bacterial cells, whose collective metabolic repertoire far exceeds that encoded by the human genome (43). Under homeostatic conditions, SCFA-producing commensals such as Fecalibacterium prausnitzii, Roseburia intestinalis, and Eubacterium rectale maintain epithelial integrity, suppress pro-inflammatory signaling, and foster mucosal immune tolerance. CRC-associated microbiomes exhibit reduced alpha-diversity and enrichment of pathobionts across geographically diverse cohorts. Two landmark cross-cohort metagenomic studies identified a reproducible core set of CRC-enriched species as reliable microbial biomarkers of CRC, including Fn, enterotoxigenic Bacteroides fragilis (ETBF), Parvimonas micra, and Peptostreptococcus anaerobius (44, 45). ETBF promotes colorectal tumorigenesis through Th17 T cell activation and downstream STAT3 signaling (46); its metalloprotease toxin fragilysin independently disrupts epithelial barrier integrity by cleaving E-cadherin, secondarily activating β-catenin and NF-κB in colonic epithelial cells. P. anaerobius drives cholesterol-dependent colonocyte proliferation and immunosuppressive myeloid cell accumulation (47).

The microbiome also modulates the pharmacokinetics and toxicity of anticancer agents, with direct clinical implications. β-Glucuronidase activity from colonic Escherichia coli and Clostridium species reactivates the toxic irinotecan metabolite SN-38, precipitating dose-limiting diarrhea that can be attenuated by microbiome-targeted strategies (48, 49). Abundance of Akkermansia muciniphila, Bifidobacterium species, and Fecalibacterium prausnitzii positively predicts anti-PD-1 responsiveness (50). Antibiotic exposure within 30 days before immunotherapy initiation has been associated with shorter progression-free survival across multiple tumor types (51)—an observation that elevates microbiome preservation to a clinical priority during immunotherapy planning. The mechanistic basis involves modulation of intratumoral CD8^+^ T cell infiltration and systemic cytokine profiles (52), with direct implications for antibiotic stewardship in oncology.

### 4.2 Perioperative probiotics

Perioperative probiotic supplementation helps restore the microbial homeostasis disrupted by mechanical bowel preparation, general anesthesia, and prophylactic antibiotics. Across 16 RCTs in patients undergoing CRC surgery, perioperative probiotic use was associated with significantly lower overall postoperative infectious complication rates (RR 0.45, 95% CI 0.27–0.76; I2 = 43%) (53). A confirmatory meta-analysis of 10 RCTs (n = 1,276) demonstrated significant reductions in diarrhea (OR 0.42, 95% CI 0.31–0.55; I2 = 28%), accelerated return of bowel function, and shortened hospitalization (54). Moderate heterogeneity in the larger meta-analysis likely reflects variation in probiotic strain composition, dosing schedules, and surgical technique across trials. Most trials employed multi-strain Lactobacillus and Bifidobacterium formulations at 109–1010 CFU/day, initiated 3–7 days preoperatively and continued 7–14 days postoperatively. Mechanisms include competitive exclusion of pathogens, mucosal IgA upregulation, and epithelial barrier reinforcement. Perioperative multi-strain probiotics may be considered as a promising adjunct within enhanced recovery after surgery (ERAS) protocols for CRC, pending comparative effectiveness trials to standardize strain selection and dosing.

### 4.3 Fecal microbiota transplantation as an immunotherapy sensitization strategy

FMT reconstitutes the recipient microbiome by transferring an intact donor microbial community. Two seminal Science trials demonstrated that FMT from anti-PD-1 responders achieved objective response rates of approximately 30% (Baruch et al.) and 20% (Davar et al.) in previously refractory melanoma patients, with mechanistic analyses confirming enhanced CD8^+^ T cell infiltration in responders (55, 56). Microsatellite-stable (MSS) CRC—representing approximately 85% of metastatic cases—exhibits near-complete primary resistance to immune checkpoint inhibitors, with pembrolizumab monotherapy response rates below 5%. This makes FMT-mediated immunotherapy sensitization an active area of investigation for MSS CRC. A phase I randomized trial of live bacterial supplementation combined with nivolumab plus ipilimumab in renal cell carcinoma established feasibility (57). CRC-specific FMT–immunotherapy trials are ongoing; current evidence does not support FMT for CRC outside approved trial frameworks.

## 5 *Fusobacterium nucleatum* in CRC: mechanisms and therapeutic implications

### 5.1 Epidemiology and prognostic significance

**Figure 1:**
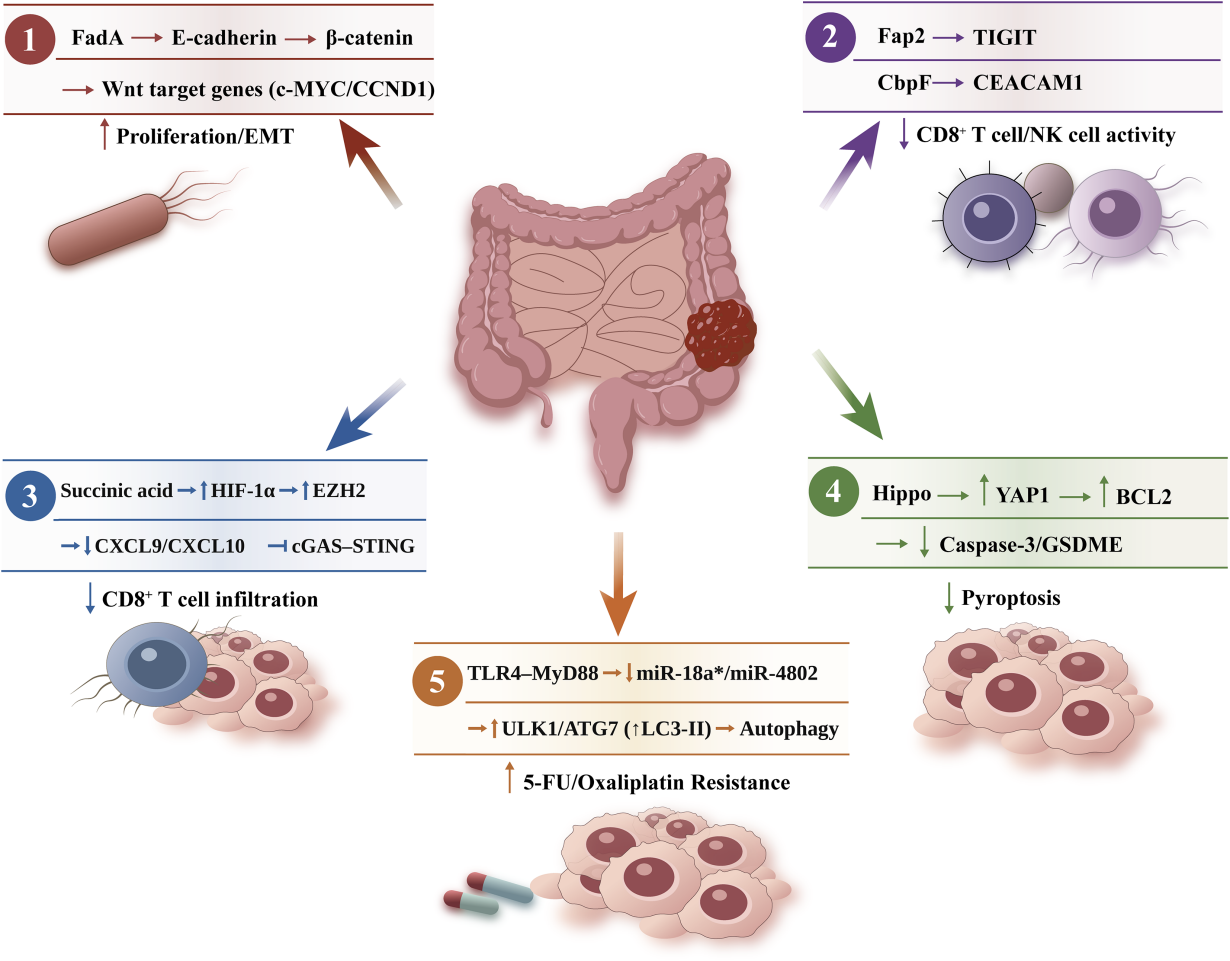
Five mechanistically distinct oncogenic pathways through which *Fusobacterium nucleatum* (Fn) promotes colorectal carcinogenesis and treatment resistance. (1) FadA-mediated Wnt/β-catenin activation. FadA binding to E-cadherin triggers β-catenin nuclear translocation, driving expression of pro-oncogenic Wnt target genes (MYC, CCND1) and promoting colonocyte proliferation and epithelial–mesenchymal transition (EMT). (2) Fap2 engagement of TIGIT and CbpF engagement of CEACAM1 mediate immune evasion by suppressing CD8^+^ T cell and NK cell cytotoxicity in the CRC tumor microenvironment. (3) Fn-derived succinic acid stabilizes HIF-1α, which upregulates the histone methyltransferase EZH2; EZH2-mediated H3K27 trimethylation epigenetically silences Th1-type chemokine and interferon-stimulated genes (e.g., CXCL9/CXCL10), while succinic acid concurrently suppresses cGAS–STING–dependent type I interferon signaling, together reducing intratumoral CD8^+^ T cell infiltration and sustaining an immunologically cold tumor microenvironment. (4) Hippo pathway activation of YAP1 upregulates BCL2 and downregulates Caspase-3 and GSDME, suppressing pyroptotic cell death and contributing to chemoresistance and immune inertness. (5) TLR4–MyD88 signaling downregulates the microRNAs miR-18a* and miR-4802, derepressing the autophagy initiators ULK1 and ATG7 (with increased LC3-II conversion) and activating autophagy, which promotes resistance to 5-fluorouracil (5-FU) and oxaliplatin.

*Fusobacterium nucleatum* (Fn) is an anaerobic gram-negative oral commensal that is overrepresented in CRC tumor tissue and has well-characterized carcinogenic mechanisms, independent prognostic significance, and several druggable molecular targets (8). Five mechanistically distinct carcinogenic pathways are reviewed below (**Figure 1**).

Intratumoral Fn is detected in 50–70% of CRC cases compared with 10–15% of adjacent normal colonic mucosa, a distribution reproduced across geographically diverse cohorts from Asia, Europe, and North America (58, 59). High intratumoral Fn abundance is associated with a specific adverse clinicopathological profile: proximal colonic location, mucinous histology, the microsatellite instability-high (MSI-H) phenotype, CpG island methylator phenotype (CIMP), and *BRAF* V600E mutation (59, 60). Oral Fn strains are genetically identical to those isolated from matched CRC tumors in the same patients, supporting hematogenous oral-to-colonic translocation; the Fap2 lectin facilitates this process by binding Gal-GalNAc moieties overexpressed on CRC cells (61). High intratumoral Fn abundance independently predicts worse overall and disease-free survival across multiple independent cohorts (59, 62), establishing Fn as a clinically relevant prognostic biomarker.

A recent pangenomic study has refined Fn subspecies taxonomy in CRC. Zepeda-Rivera et al. generated closed genomes from 135 Fn strains (80 oral strains from individuals without cancer and 55 strains cultured from tumors in 51 CRC patients) and identified 483 CRC-enriched genetic factors. Fn subspecies animalis (Fna)—previously treated as a single subspecies—was shown to comprise two distinct clades: Fna C1, largely confined to the oral cavity, and Fna C2, which dominates the CRC tumor niche. Fna C2 harbors additional virulence factors including fap2 and fusolisin, displays distinct morphology, and possesses enhanced metabolic capacity for gastrointestinal colonization. In Apc^Min/+^ mice, Fna C2 gavage increased the number of intestinal adenomas and altered metabolite profiles. Microbiome analysis of tumor tissue from 116 CRC patients further demonstrated Fna C2 enrichment, and direct comparison of 62 paired specimens showed that only Fna C2 was enriched in tumor tissue relative to adjacent normal mucosa; metagenomic analysis of stool from 627 CRC patients and 619 healthy individuals provided independent support. Collectively, these results establish that Fna C2—rather than Fn as a whole—is the relevant target for mechanistic and therapeutic investigation in CRC (63).

### 5.2 FadA-mediated Wnt/β-catenin activation

Among Fn virulence factors, the FadA adhesin is the most mechanistically characterized effector in CRC. FadA binds E-cadherin on colonocyte surfaces with high affinity, triggering β-catenin release from the adherens junction complex and its nuclear translocation. In the nucleus, β-catenin associates with TCF/LEF transcription factors to drive expression of pro-proliferative targets including *MYC*, cyclin D1 (*CCND1*), and *AXIN2*, established oncogenic drivers in CRC (64). Approximately 70–80% of CRCs already harbor *APC* mutations that constitutively activate Wnt/β-catenin signaling; FadA therefore superimposes a microorganism-derived activation stimulus on an already dysregulated pathway. Fn also potentiates intestinal tumorigenesis through remodeling of the tumor–immune microenvironment (65). Small-molecule inhibitors of the FadA–E-cadherin interaction reduce β-catenin nuclear translocation and tumor growth in Fn-colonized CRC organoid models, identifying FadA as a candidate therapeutic target at the preclinical stage.

### 5.3 Fap2- and CbpF-mediated immune evasion via TIGIT and CEACAM1

Fap2, the second major Fn virulence lectin, mediates both tumor colonization through Gal-GalNAc binding (61) and immune evasion by directly engaging inhibitory checkpoint receptors, notably T cell immunoreceptor with Ig and ITIM domains (TIGIT) and carcinoembryonic antigen-related cell adhesion molecule 1 (CEACAM1). Fap2–TIGIT engagement triggers immunoreceptor tyrosine-based inhibitory motif (ITIM)-mediated recruitment of SHP-1 and SHP-2 phosphatases, which dephosphorylate activating kinases and thereby suppress NK cytotoxicity, T cell interferon-γ secretion, and perforin-mediated cytolysis (66, 67). Fn also engages CEACAM1 through the trimeric autotransporter adhesin CbpF, broadening its immune-evasion capacity beyond the TIGIT axis (68, 69). Neutralizing antibodies blocking the Fap2–TIGIT interaction restore NK cell-mediated antitumor killing *in vitro* and reduce tumor burden in humanized xenograft models (66). In MSS CRC, Fn-mediated engagement of these checkpoint receptors provides a plausible mechanistic basis for immune checkpoint resistance.

### 5.4 Succinate–HIF-1α–EZH2-mediated immune suppression

The third Fn-mediated carcinogenic pathway involves reprogramming of host colonocyte metabolism to dampen antitumor immunity. Fn infection disrupts tricarboxylic acid cycle flux and impairs succinate dehydrogenase (SDH) activity, resulting in intracellular accumulation of succinate, which is also released as Fn-derived succinic acid (70). Accumulated succinate stabilizes HIF-1α under normoxic conditions by competitively inhibiting prolyl hydroxylase domain enzymes. Stabilized HIF-1α upregulates the polycomb histone methyltransferase EZH2, whose H3K27 trimethylation epigenetically silences the interferon-stimulated and Th1-type chemokine genes that govern cytotoxic T cell recruitment. In parallel, Fn-derived succinic acid suppresses cGAS–STING–dependent type I interferon signaling at the functional level, rather than by repressing STING1 gene transcription (70, 71). The net consequence is reduced intratumoral CD8^+^ T cell infiltration and an immunologically cold tumor microenvironment characteristic of MSS CRC and checkpoint-inhibitor resistance. This succinate–HIF-1α–EZH2 axis is pharmacologically accessible at multiple nodes: Fn eradication to prevent succinate accumulation, EZH2 inhibition to restore chemokine expression and effector T cell recruitment, or STING agonism to bypass interferon suppression, individually or in combination as an immunotherapy-sensitization strategy.

### 5.5 Hippo pathway-mediated suppression of pyroptosis

Within CRC cells, Fn infection activates YAP1 (Yes-associated protein 1), the transcriptional effector of the Hippo pathway. YAP1 upregulates BCL2 while concurrently suppressing Caspase-3 and Gasdermin E (GSDME), the key executors of pyroptotic cell death (72). By dampening pyroptosis, Fn-mediated YAP1 activation reduces chemotherapy sensitivity and promotes an immunologically inert tumor microenvironment. YAP1 inhibition and restoration of GSDME-mediated pyroptosis thus represent candidate therapeutic targets in Fn-colonized CRC (71, 72).

### 5.6 Autophagy induction and chemoresistance

Fn engages TLR4–MyD88 innate immune signaling in CRC cells. Yu et al. demonstrated that this signaling selectively downregulates two host microRNAs, miR-18a* and miR-4802, which normally restrain the autophagy initiators ULK1 and ATG7; loss of these microRNAs therefore derepresses ULK1 and ATG7 and activates the autophagy pathway, accompanied by increased LC3-II conversion. The resulting autophagic flux blunts apoptotic responses to 5-fluorouracil and oxaliplatin and promotes chemotherapy-resistant cell survival. Either pharmacological autophagy inhibition with chloroquine or hydroxychloroquine, or restoration of miR-18a* and miR-4802, resensitized Fn-infected CRC cells to chemotherapy *in vitro* and *in vivo* (73). A separate study found that intratumoral Fn abundance differentially modulates immune responses according to microsatellite instability status, placing the autophagy-driven resistance mechanism within the broader context of Fn-mediated immune remodeling (74). These findings support testing autophagy inhibitor combinations in Fn-high CRC as a strategy to restore chemosensitivity.

### 5.7 Therapeutic targeting of *Fusobacterium nucleatum*

All Fn-targeted therapeutic strategies described below remain at the preclinical or early investigational stage; none has demonstrated efficacy in human RCTs, and no Fn-directed therapy is currently approved for routine CRC care. The identification of Fna C2 as the dominant CRC-associated clade (63) indicates that future therapeutic strategies should target this specific lineage. Several Fn-targeted strategies are under active investigation. Metronidazole efficiently eradicates Fn in preclinical models, and retrospective clinical analyses document reduced intratumoral Fn abundance and improved outcomes in patients receiving metronidazole-containing regimens (75). Broad-spectrum antibiotic use, however, carries attendant risks of collateral microbiome disruption, antimicrobial resistance selection, and attenuation of immunotherapy efficacy. More selective approaches include small-molecule FadA–E-cadherin inhibitors (64), Fap2–TIGIT blocking antibodies (66), and probiotic preparations that competitively suppress Fn colonization (53). Bacteriophage therapy is emerging as a candidate approach for species-specific Fn elimination without collateral microbiome disruption, though human clinical data remain absent (71). Standardized quantitative PCR of tumor tissue or stool—preferably Fna C2-specific where the assay is available—is the prerequisite detection methodology for patient stratification in future Fn-targeted trials (8, 63).

## 6 Mind-body interventions in CRC survivorship

### 6.1 SIO–ASCO and ASCO–SIO clinical practice guidelines (2022–2024)

CRC survivors face a distinctive set of long-term sequelae beyond the anxiety, depression, fatigue, and fear of recurrence common to cancer survivors generally. Bowel dysfunction after anterior resection or permanent stoma formation, oxaliplatin-induced peripheral neuropathy, sexual dysfunction following pelvic surgery or radiotherapy, and body image concerns related to ostomy appliances impose burdens largely specific to this patient population (4, 76). Three successive SIO–ASCO and ASCO–SIO clinical practice guidelines issued between 2022 and 2024 provide evidence-graded recommendations for integrative survivorship interventions (9–11), establishing a framework for systematically incorporating mind-body medicine and acupuncture into standard cancer care.

The 2022 SIO–ASCO Guideline on Integrative Medicine for Pain Management in Oncology—informed by a systematic review of RCTs—recommends acupuncture for musculoskeletal cancer pain and aromatase inhibitor-related arthralgia (9). The 2023 SIO–ASCO Guideline on Anxiety and Depression in Cancer identifies mindfulness-based interventions (MBIs) as the strongest recommendation for both anxiety and depression during active treatment and post-treatment, with yoga receiving strong recommendations for anxiety and depression management during active cancer treatment (10). The 2024 ASCO–SIO Fatigue Management Guideline Update assigns strong recommendations to exercise, MBIs, and cognitive behavioral therapy (CBT) for cancer-related fatigue during and after treatment completion, with yoga receiving a conditional recommendation for post-treatment fatigue (11). Effect sizes across these modalities typically fall in the small-to-moderate range (standardized mean difference [SMD] 0.3–0.6). While broadly comparable to pharmacological comparators for cancer-related symptom management, these benefits are achieved with a substantially more favorable safety profile.

### 6.2 Mindfulness-based interventions and yoga

Mindfulness-based stress reduction (MBSR) and mindfulness-based cognitive therapy (MBCT) are structured 8-week programs comprising weekly 2.5-hour group sessions supplemented by daily home meditation practice. Ng et al. reported clinically meaningful improvements in anxiety (SMD −0.51), depression (SMD −0.56), and quality of life (SMD 0.39) across multiple cancer types, with effects maintained at 6-month follow-up (77). Haller et al. subsequently identified intervention fidelity and participant adherence as key moderators of effect size (78). MBIs attenuate hypothalamic–pituitary–adrenal (HPA) axis reactivity, enhance parasympathetic tone—evidenced by improved heart-rate variability—and reduce circulating IL-6 and C-reactive protein. The available evidence derives predominantly from breast cancer and mixed-cancer populations; while biologically plausible for CRC, direct extrapolation should be made with caution given the distinct symptom profile of CRC survivors (bowel dysfunction, stoma-related concerns, oxaliplatin-induced neuropathy). CRC-specific RCTs are needed to validate these findings in CRC populations.

Yoga combines physical postures (asanas), controlled breathing (pranayama), and meditative awareness. It produces multidimensional benefits in survivor populations. In a meta-analysis of RCTs, yoga practice yielded significant improvements in fatigue (SMD −0.48), quality of life (SMD 0.42), anxiety (SMD −0.41), depression (SMD −0.49), and sleep quality (SMD 0.38) (79). Standard delivery involves 60–90-minute sessions two to three times weekly over 8–12 weeks, with adverse event rates below 2% when programs are appropriately adapted for cancer survivors. As with MBIs, evidence derives predominantly from breast cancer trials, and CRC-specific data are needed.

### 6.3 Acupuncture

Among integrative interventions for cancer-related pain, acupuncture has the most extensive evidence base. Chiu et al. reported significant pain reduction relative to usual care or sham acupuncture (SMD −0.58, 95% CI −0.80 to −0.36) across post-surgical, neuropathic, and musculoskeletal pain subtypes (80), with effect sizes comparable to those of NSAIDs and weak opioids in non-cancer pain populations and adverse event rates below 3%.

Chemotherapy-induced peripheral neuropathy (CIPN) affects up to 70% of CRC patients receiving FOLFOX-based regimens and currently lacks any pharmacological treatment of established efficacy. A randomized controlled pilot trial demonstrated significant improvements in neuropathy severity, sensory function, and quality of life in the acupuncture arm, with benefits persisting beyond the active treatment period (81). One caveat deserves emphasis: existing RCT evidence for acupuncture in CIPN derives predominantly from paclitaxel-treated breast cancer patients, not from oxaliplatin-treated CRC patients. Paclitaxel-induced neuropathy (microtubule disruption, dorsal root ganglion toxicity) and oxaliplatin-induced neuropathy (platinum–DNA adducts, acute cold allodynia via Nav1.6 channel sensitization) differ substantially in their underlying mechanisms, and clinicians should discuss this evidence gap transparently when recommending acupuncture for oxaliplatin-induced CIPN.

## 7 Discussion

Evidence-based integrative strategies now span every stage of the CRC care continuum, from primary chemoprevention through long-term survivorship (**Table 1**). High-fiber diets, Mediterranean dietary patterns, calcium supplementation, regular physical activity, healthy weight maintenance, and berberine each provide clinically meaningful CRC risk reduction (6, 7, 14, 19, 24, 25, 30, 31). The CHALLENGE trial now provides the first randomized phase 3 evidence that structured exercise after adjuvant chemotherapy reduces disease recurrence and death in colon cancer, confirming what observational data had long suggested (29). Aspirin chemoprevention should be guided by the 2022 USPSTF recommendations with individualized benefit–risk assessment (33, 35). Preliminary conference data from CaPP3 are consistent with low-dose aspirin efficacy in Lynch syndrome (34); however, these findings have not yet undergone peer review, and clinical recommendations based on them remain provisional pending formal publication.

**Table 1:** Summary of key integrative oncology interventions across the colorectal cancer care continuum.

Intervention	Category	Clinical phase	Mechanism of action	Effect size / key outcome	Evidence level	Clinical recommendation [Ref.]
Dietary and lifestyle interventions						
High-fiber diet (≥25–30 g/day)	Dietary	Prevention	Butyrate-mediated HDAC inhibition; NF-κB suppression; tight-junction upregulation; mucosal barrier reinforcement	~10% lower CRC risk per 10 g/day fiber increment; dose–response meta-analysis, n > 2 million	High	Recommend ≥25–30 g/day for all adults at average or elevated CRC risk (6, 14)
Mediterranean diet	Dietary pattern	Prevention	NF-κB suppression via olive oil polyphenols; COX-2/PGE2 attenuation via omega-3 fatty acids; SCFA-producing microbiota enrichment; TMAO reduction	RR 0.86 (95% CI 0.80–0.94) vs. lowest adherence; corroborated in 117-study meta-analysis, n = 3.2 million	High	Recommend as cornerstone of CRC prevention (19, 20)
Calcium supplementation (1,000–1,200 mg/day)	Dietary supplement	Prevention	Luminal binding of secondary bile acids; CaSR-mediated epithelial differentiation and antiproliferation	RR 0.80 (95% CI 0.68–0.93) for colorectal adenoma recurrence	High	Recommend for patients with prior colorectal adenoma resection (6, 16)
Vitamin D supplementation	Dietary supplement	Prevention	VDR-mediated regulation of cell cycle, apoptosis, and immune surveillance	OR 0.88 per 25 nmol/L increment, Mendelian randomization; null result in VITAL RCT	Moderate	Correct documented deficiency; routine supplementation not supported by current RCT evidence (17, 18)
Red/processed meat reduction	Dietary	Prevention	Reduced N-nitroso compounds, heme iron-catalyzed lipid peroxidation, and heterocyclic amines	RR 1.17 per 100 g/day red meat (95% CI 1.05–1.31); RR 1.18 per 50 g/day processed meat (95% CI 1.10–1.28)	High	Limit red meat to <500 g cooked/week; minimize processed meat (22)
Alcohol reduction/abstinence	Lifestyle	Prevention	Reduced acetaldehyde-induced DNA damage; restored folate-mediated one-carbon metabolism	RR 1.44 for heavy drinking ≥50 g ethanol/day vs. nondrinkers and occasional drinkers; continuous dose–risk relationship	High	Minimize alcohol; abstinence preferred for high-risk individuals (23)
Regular physical activity (≥150 min/week moderate intensity)	Lifestyle	Prevention / Survivorship	Reduced insulin/IGF-1; attenuation of adipokine-driven inflammation; COX-2 downregulation; accelerated colonic transit	Summary RR 0.76 (95% CI 0.72–0.81) for colon cancer, most vs. least active; HR 0.61 (95% CI 0.40–0.92) for CRC-specific mortality post-diagnosis; CHALLENGE trial (NEJM 2025): HR 0.72 (95% CI 0.55–0.94) for DFS, HR 0.63 (95% CI 0.43–0.94) for OS after adjuvant chemotherapy	High	≥150 min/week moderate aerobic + 2×/week resistance training; interrupt prolonged sitting (24, 25, 29)
Healthy weight maintenance (BMI 18.5–24.9)	Lifestyle	Prevention	Reduced hyperinsulinemia; lower TNF-α/IL-6/leptin; PI3K/AKT/mTOR downregulation; normalized adiponectin signaling	RR 1.24 per 5-unit BMI increment in men (95% CI 1.20–1.28); visceral adiposity independently increases risk	High	Maintain healthy BMI; reduce waist circumference to mitigate visceral adiposity risk (30, 31)
Chemoprevention and phytochemicals						
Aspirin (75–300 mg/day)	Chemoprevention	Prevention	Irreversible COX-2 inhibition; PGE2 suppression; reduced pro-angiogenic and pro-proliferative signaling	20–30% lower CRC incidence with ≥10-yr use; CAPP2 Lynch syndrome: ITT HR 0.65 (0.43–0.97), per-protocol HR 0.56 (0.34–0.91); CaPP3 2025 conference^§^: 75–100 mg/day non-inferior to 600 mg/day in Lynch syndrome	High (Lynch) Moderate (avg. risk)	USPSTF Grade D ≥60 yr; individualized assessment age 40–59 yr; low-dose may be considered in Lynch syndrome based on CAPP2 RCT evidence; preliminary CaPP3 conference data^§^ remain provisional pending peer-reviewed publication (32–35)
Berberine (0.3 g twice daily)	Phytochemical	Prevention (secondary)	AMPK activation; mTOR suppression; COX-2 inhibition; gut microbiome remodeling	RR 0.77 (95% CI 0.66–0.91); NNT ≈ 9; benefit suggested at 6-year retrospective follow-up	High	May be considered (0.3 g twice daily) for secondary prevention after adenoma resection based on the CBAR trial; international confirmatory trials in non-Chinese populations needed before broad guideline endorsement (7, 36)
Curcumin	Phytochemical	Investigational	NF-κB, Wnt/β-catenin, STAT3, and EGFR pathway inhibition	60.4% polyp reduction with curcumin + quercetin in uncontrolled FAP pilot (n = 5; not attributable to curcumin alone); no efficacy signal in phase IIa RCT	Low	Insufficient evidence—research only; bioavailability-optimized formulations require CRC-specific phase III RCT validation (39, 41)
Microbiome-targeted interventions						
*Fusobacterium nucleatum*-targeted therapy	Microbiome-targeted	Investigational	FadA–E-cadherin inhibition (Wnt/β-catenin); Fap2–TIGIT and CbpF–CEACAM1 immune checkpoint blockade; succinate–HIF-1α–EZH2 axis targeting (EZH2 inhibition); Hippo/YAP1–pyroptosis restoration; autophagy inhibition (miR-18a*/miR-4802–ULK1/ATG7 axis)	Preclinical: metronidazole reduces intratumoral Fn; FadA inhibitors, Fap2–TIGIT antibodies, bacteriophage under investigation	Low (preclinical)	Preclinical/investigational only; no approved Fn-targeted therapy for routine CRC use. qPCR-based Fn quantification (preferably Fna C2-specific) required for patient stratification in future trials (8, 63, 64, 66, 68–70, 72, 73, 75)
Perioperative multi-strain probiotics (*Lactobacillus* + *Bifidobacterium*, 10^9^–10^10^ CFU/day)	Microbiome-targeted	Active treatment (perioperative)	Competitive exclusion of pathogens; mucosal IgA upregulation; epithelial barrier reinforcement	RR 0.45 (95% CI 0.27–0.76; I^2^ = 43%) for postoperative infectious complications, 16 RCTs; OR 0.42 (95% CI 0.31–0.55; I^2^ = 28%) for diarrhea, 10 RCTs	Moderate	May be considered for incorporation into ERAS protocols (initiate 3–7 days preoperatively, continue 7–14 days postoperatively); pending comparative effectiveness trials to standardize strain selection and dosing (53, 54)
Fecal microbiota transplantation (FMT)	Microbiome-targeted	Investigational	Donor microbiome reconstitution; enhanced CD8^+^ T cell infiltration; restored anti-PD-1 responsiveness	Objective response rates of ~30% (Baruch et al.) and ~20% (Davar et al.) in anti-PD-1-refractory melanoma; no CRC-specific RCT data available	Low	Approved clinical trial frameworks only; not for routine CRC use outside trials (55, 56)
Mind-body interventions						
Mindfulness-based stress reduction/MBCT (8-week programs)	Mind-body	Active treatment / Survivorship	HPA-axis reactivity attenuation; parasympathetic tone enhancement; IL-6 and CRP reduction	Standardized mean difference (SMD) −0.51 (anxiety); −0.56 (depression); 0.39 (QoL); effects maintained at 6-month follow-up; evidence predominantly from breast cancer and mixed-cancer populations	High	8-week MBSR/MBCT for anxiety, depression, and QoL impairment; evidence derives from mixed-cancer populations; CRC-specific RCTs needed (10, 77)
Yoga (60–90 min, 2–3×/week, 8–12 weeks)	Mind-body	Active treatment / Survivorship	HPA-axis modulation; parasympathetic activation; reversal of physical deconditioning	SMD −0.48 (fatigue); 0.42 (QoL); −0.41 (anxiety); −0.49 (depression); 0.38 (sleep quality)	High (anxiety/depression Rx) Moderate (post-Rx fatigue)	May be considered for anxiety and depression during active treatment; conditional for post-treatment fatigue; evidence from mixed-cancer populations; CRC-specific trials needed (10, 11, 79)
Acupuncture	Integrative procedure	Active treatment / Survivorship	Endogenous opioid activation; descending pain inhibitory pathway modulation; adenosine-mediated anti-inflammatory effects	SMD −0.58 (95% CI −0.80 – −0.36) for cancer-related pain vs. usual care or sham; significant CIPN improvement in pilot randomized controlled trial (paclitaxel-treated population)	High (cancer pain) Moderate (CIPN)	Recommend for musculoskeletal cancer pain (9); consider for CIPN with explicit acknowledgment that oxaliplatin-specific randomized controlled trial evidence is lacking (80, 81)

CaSR, calcium-sensing receptor; CIPN, chemotherapy-induced peripheral neuropathy; CRC, colorectal cancer; ERAS, enhanced recovery after surgery; FAP, familial adenomatous polyposis; HDAC, histone deacetylase; HPA, hypothalamic–pituitary–adrenal; MBCT, mindfulness-based cognitive therapy; MBSR, mindfulness-based stress reduction; NNT, number needed to treat; QoL, quality of life; SMD, standardized mean difference; TMAO, trimethylamine N-oxide; USPSTF, US Preventive Services Task Force; VDR, vitamin D receptor. Evidence levels: (High) consistent evidence from ≥2 randomized controlled trials or meta-analyses; (Moderate) fewer or methodologically limited randomized controlled trials or cohort studies; (Low) preclinical studies, retrospective analyses, or small uncontrolled trials. ^§^Preliminary data presented at the Cancer Research UK International Cancer Prevention Conference, London, 25–27 June 2025; not yet published in peer-reviewed form at the time of submission. As conference data, these findings should be treated as provisional pending formal peer-reviewed publication.

*Fusobacterium nucleatum* has accumulated sufficient prognostic evidence to merit consideration as a biomarker in CRC clinical assessment, while its therapeutic targeting remains at the preclinical stage. Five mechanistically distinct carcinogenic pathways position Fn as a clinically relevant oncogenic driver with multiple therapeutic entry points: FadA-mediated Wnt/β-catenin activation, Fap2- and CbpF-mediated immune evasion via TIGIT and CEACAM1, succinate–HIF-1α–EZH2 immune suppression, Hippo pathway-mediated suppression of pyroptosis, and autophagy-induced chemoresistance (64, 66, 68–70, 72, 73). The discovery that specifically the Fna C2 clade dominates the CRC tumor niche (63) refines this framework and directs future research toward clade-level precision. Standardized PCR-based quantification of intratumoral or stool-derived Fn would enable patient stratification within precision oncology frameworks (8). Perioperative multi-strain probiotics may be considered as a promising adjunct within ERAS protocols based on RCT meta-analytic evidence, pending standardization of strain selection and dosing (53, 54).

Translating this evidence into clinical practice requires systematic distress screening, accessible referral pathways, and health-system recognition of mindfulness-based interventions, yoga, and acupuncture as reimbursable components of comprehensive oncology care—all endorsed by the three successive SIO–ASCO and ASCO–SIO clinical practice guidelines (2022–2024).

### 7.1 Limitations

Several limitations merit explicit acknowledgment. As a narrative rather than systematic review, study selection was subject to the authors’ judgment and was not governed by a formal protocol or PRISMA process. A substantial portion of the evidence for MBIs, yoga, and acupuncture derives from breast cancer populations, and extrapolation to CRC survivors should be made with caution. Chemoprevention data for berberine derive entirely from Chinese hospital-based cohorts, and generalizability to non-Chinese populations has not yet been established. Acupuncture evidence for CIPN is predominantly from paclitaxel-treated patients, and mechanistic differences between paclitaxel- and oxaliplatin-induced neuropathy limit direct application to CRC populations. The CaPP3 trial data were presented at a conference in June 2025 and have not undergone formal peer review at the time of this writing; clinical recommendations based on these findings should therefore be treated as provisional. The rapidly evolving microbiome literature means that some findings discussed here may be superseded by data from ongoing trials.

### 7.2 Future research priorities

Four research priorities emerge from this review. First, adequately powered RCTs of microbiome-targeted therapies with survival endpoints are urgently needed, including FMT for MSS CRC immunotherapy sensitization and Fn-directed interventions. Second, bioavailability-optimized curcumin formulations require CRC-specific phase II/III RCT validation before clinical adoption. Third, CRC-specific RCTs of acupuncture for oxaliplatin-induced CIPN, and of MBIs and yoga in CRC survivor populations, are needed to move beyond extrapolation from breast cancer cohorts. Fourth, implementation-science research must address structural barriers that impede equitable delivery of these interventions, including geographic access disparities, inadequate insurance coverage, and variable clinician awareness.

## Glossary

AMPK: AMP-activated protein kinase
BCL2: B-cell lymphoma 2 protein
CaSR: calcium-sensing receptor
CEACAM1: carcinoembryonic antigen-related cell adhesion molecule 1
cGAS: cyclic GMP–AMP synthase
CIMP: CpG island methylator phenotype
CIPN: chemotherapy-induced peripheral neuropathy
COX-2: cyclooxygenase-2
CRC: colorectal cancer
ERAS: enhanced recovery after surgery
ETBF: enterotoxigenic *Bacteroides fragilis*
EZH2: enhancer of zeste homolog 2
FMT: fecal microbiota transplantation
Fn: *Fusobacterium nucleatum*
GSDME: Gasdermin E
H3K27me3: trimethylation of histone H3 at lysine 27
HDAC: histone deacetylase
HIF-1α: hypoxia-inducible factor 1-alpha
HPA: hypothalamic–pituitary–adrenal
IGF-1: insulin-like growth factor 1
MBCT: mindfulness-based cognitive therapy
MBIs: mindfulness-based interventions
MBSR: mindfulness-based stress reduction
MSI-H: microsatellite instability-high
MSS: microsatellite-stable
NNT: number needed to treat
PGE2: prostaglandin E2
QoL: quality of life
RCT: randomized controlled trial
SCFAs: short-chain fatty acids
SDH: succinate dehydrogenase
SMD: standardized mean difference
STING: stimulator of interferon genes
TIGIT: T cell immunoreceptor with Ig and ITIM domains
TMAO: trimethylamine N-oxide
USPSTF: US Preventive Services Task Force
VDR: vitamin D receptor
YAP1: Yes-associated protein 1.
